# Persistent Fluctuations in Stride Intervals under Fractal Auditory Stimulation

**DOI:** 10.1371/journal.pone.0091949

**Published:** 2014-03-20

**Authors:** Vivien Marmelat, Kjerstin Torre, Peter J. Beek, Andreas Daffertshofer

**Affiliations:** 1 Movement to Health Laboratory, Montpellier-1 University, EuroMov, Montpellier, France; 2 MOVE Research Institute Amsterdam, Faculty of Human Movement Sciences, VU University Amsterdam, Amsterdam, Netherlands; 3 School for Sport and Education, Brunel University, Uxbridge, Middlesex, United Kingdom; University of Toronto, Italy

## Abstract

Stride sequences of healthy gait are characterized by persistent long-range correlations, which become anti-persistent in the presence of an isochronous metronome. The latter phenomenon is of particular interest because auditory cueing is generally considered to reduce stride variability and may hence be beneficial for stabilizing gait. Complex systems tend to match their correlation structure when synchronizing. In gait training, can one capitalize on this tendency by using a fractal metronome rather than an isochronous one? We examined whether auditory cues with fractal variations in inter-beat intervals yield similar fractal inter-stride interval variability as isochronous auditory cueing in two complementary experiments. In Experiment 1, participants walked on a treadmill while being paced by either an isochronous or a fractal metronome with different variation strengths between beats in order to test whether participants managed to synchronize with a fractal metronome and to determine the necessary amount of variability for participants to switch from anti-persistent to persistent inter-stride intervals. Participants did synchronize with the metronome despite its fractal randomness. The corresponding coefficient of variation of inter-beat intervals was fixed in Experiment 2, in which participants walked on a treadmill while being paced by non-isochronous metronomes with different scaling exponents. As expected, inter-stride intervals showed persistent correlations similar to self-paced walking only when cueing contained persistent correlations. Our results open up a new window to optimize rhythmic auditory cueing for gait stabilization by integrating fractal fluctuations in the inter-beat intervals.

## Introduction

The assessment of mean and standard deviation alone often does not suffice to discriminate between optimal and constrained behavior or between healthy and pathological performances [1]–[3]. The temporal structure of fluctuations, here synonym for the serial-lag correlation of consecutive events, may contain valuable information about the functional organization of the system generating these events [4]–[6]. The presence of long-range correlations or *1/f noise*, be it in cortical activity, EMG activity, or macroscopic gait dynamics, is considered a generic marker for systems that can adequately adapt to perturbations in their environment [5], [7]–[9]. Many clinical studies revealed dependencies of the correlation structure of gait to different pathologies like amyotrophic lateral sclerosis [1], Huntington’s disease [1]–[2], and Parkinson’s disease [1], [9].

External cueing may alter the temporal correlation structure of gait. Isochronous auditory cues are particularly known for changing the typical fractal dynamics of healthy gait [10]–[11]. Persistent (positive) long-range correlations in stride intervals of self-paced gait may switch to anti-persistent (negative) correlations if an isochronous metronome is present [12]. This qualitative change of gait dynamics may be indicative of ‘local’ (i.e. short-term) coupling processes, allowing for cycle-by-cycle entrainment of the movements with the metronome. Isochronous pacing thus constrains the locomotor system to (a narrow band around) the isolated metronome frequency, which is contrary to self-paced walking where the locomotion covers a broad range of frequencies with a power-law distribution [9], [12]–[13]. In spite of this constraining feature, the beneficial capacity of isochronous auditory cueing for gait rehabilitation in the presence of neurodegenerative diseases has been demonstrated in several studies. For instance, stride length, cadence, and speed all increase [14], whereas inter-stride variability and occurrence of freezing decrease (see, e.g., [2], and for a systematic review [15]). Could this beneficial capacity be amplified if cueing contains variability similar to that of healthy gait? And does the presence of fractality in the cueing streams prepare walkers to cope with potential future irregularities in the natural environment?

Introducing variability and fractality in cueing streams is not new. Kaipust and co-workers [16] submitted that isochronous cueing might not be optimal for gait rehabilitation. They showed that participants were sensitive to the correlation structure of cueing fluctuations. However, their participants were not instructed to synchronize their gait to the metronome. It might be that subjects just ignored the metronome and fell back to their own, fractal gait structure, which would question the effect of fractal cueing. Hove and co-workers [17] showed that the correlation structure of stride times in patients suffering Parkinson’s disease changed towards that of healthy gait when using an interactive rhythmic auditory stimulation, i.e. when stimulus timing changed in response to the participant’s instant tempo. However, why this adjustment occurred remains unclear. Was it because the patients synchronized to the stimulus or vice versa?

We investigated the effect of fractal cueing when subjects were explicitly asked to synchronize with the metronome. We hypothesized that, unlike isochronous pacing, persistent long-range correlated auditory cueing preserves the fractal dynamics of stride intervals in healthy subjects. To test this hypothesis, we conducted two complementary experiments in which we measured inter-stride intervals (ISIs). In Experiment 1 participants walked when paced by either isochronous or fractal cues with different inter-beat interval (IBIs) coefficients of variation to determine the ‘optimal’ amount of variation in the cueing. In Experiment 2 participants walked when paced by either isochronous or non-isochronous cues with different scaling exponents characterizing the IBIs’s correlation structure. We expected that subjects would synchronize with any metronome but we expected stride intervals to present persistent, long-range correlations only when cues resemble the fractal fluctuations present in voluntary, self-paced walking.

### Assessing the Correlation Structure

Central to our data analysis are estimates of the scaling behavior of serial-lag correlations that we briefly summarize before outlining our experimental approach. We employed the detrended fluctuations analysis (DFA) [18], which was deemed suitable here in view of its applicability to relatively short time series [19]. DFA assesses the relationship between the magnitude of fluctuations of the variable and the duration over which these fluctuations are observed. If the correlation structure is scale free (fractal), then this relationship should obey the form
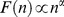
(1)where *F* denotes the fluctuation strength and *n* is a time interval that provides a measure of the aforementioned duration. The scaling exponent *α* is of essential interest: a fully random series (white noise) corresponds to *α* = 0.5; time series containing anti-persistent correlations have *α*<0.5, and persistent correlations imply *α*>0.5. The exponent *α* should be bounded to the interval [0,1] because otherwise the time series under study is non-stationary rendering subsequent, conventional statistics invalid. We also note that *α* is closely related to the Hurst exponent that is often used to characterize fractal stochastic processes – *α* equals the Hurst exponent if the to-be-analyzed time series has been generated by stationary series, i.e. fractal Gaussian noise.

### Detrended Fluctuation Analysis

**Figure 1:**
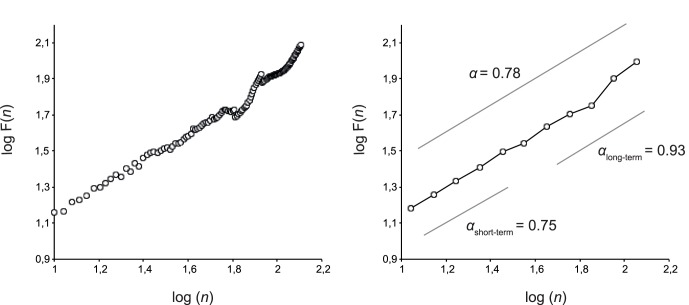
*Left panel:* Representative example of the average diffusion plots in bilogarithmic coordinates, obtained by performing DFA on inter-stride interval series (condition *CV* 1%). The average plots are computed by point-by-point averaging over the twelve participants performing under the same experimental condition. *Right panel:* Corresponding plot obtained with the 11-point averaging method. Solid line corresponds to the slope for the 11 points (*α*), the dashed lines correspond to the slope of the short-term (*α*_short-term_) and long-term (*α*_long-term_) regions of the diffusion plot.

To obtain the scaling exponent *α* one first integrates the mean-centered time series under study, which reads for the discrete time series *Y*:
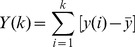
(2)where *N* corresponds to the number of samples in the series. Then, the integrated time series is divided into non-overlapping intervals of *n* data points (here 10<*n*<*N*/2). Within every interval the time series 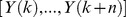 is fitted by a line 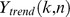, which can be interpreted as a linear, local trend. That trend is subsequently removed yielding per interval of length *n*, the mean characteristic magnitude of fluctuation *F*(*n*) as



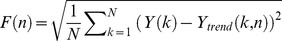
(3)As log(*n^α^*) = *α*⋅log(*n*), the scaling exponent *α* can be estimated by a slope of the diffusion plot, i.e. the log-log plot of *F* as a function of *n* (Figure 1, left panel) – we note that when determining *α* on that logarithmic scale, *n* should be sampled exponentially in order to avoid a bias in the fit towards larger *n*-values (Figure 1, right panel).

### Short-term and Long-term Correlations

We expected what may be referred to as ‘complexity matching’: when two complex systems interact, they are likely to entrain [20]–[22]. That is, we expected a strong correlation between fractal exponents of ISIs and IBIs. Because complexity matching is more likely to be manifest on long-term scales [23], we also examined the short- and long-term regions of the diffusion plots, in addition to the overall scaling exponent *α* (Figure 1, right panel). While the precise separation of short- and long-term regions might be somewhat arbitrary, one may state as a rule of thumb that short-term fluctuations account for local (brief) adaptations, whereas long-term fluctuations reveal more global (durable) changes, possibly in coordination. To guarantee proper sample sizes for reliable exponent estimates, we defined *α*_short-term_ as the slope of the first half of the diffusion plot and *α*_long-term_ as the slope of the second half. More precisely, *α*_short-term_ and *α*_long-term_ included *F*(*n*)-values over the intervals *n* = 10 … 31 and *n* = 50 … 128, respectively, for time series with 256 data-points. Note that *α*_short-term_ does not refer to short-term gait events, e.g., one or two strides, but to the length of the time series under study.

## Experiment 1 – Determining the Optimal Coefficient of Variation

We used an isochronous and a set of fractal auditory pacing signals that differed in fluctuation strength (the coefficients of variation of the inter-beat interval were 0.5%, 1%, 1.5%, and 2%, respectively) to determine the conditions in which subjects gait was influenced by the presence of fractal cues, here synonym of cues with persistent, long-range correlations.

### Methods

#### Participants

After giving written informed consent, twelve healthy volunteers (seven female, age = 28±6 years) participated in the experiment.

#### Ethics

The ethics committee of the Faculty of Human Movement Sciences, VU University Amsterdam, approved the experiment prior to its conductance.

#### Apparatus and equipment

Participants walked on a treadmill in which a single large force platform was embedded (ForceLink, Culemborg, The Netherlands), allowing for online detection of foot contact [24]. Computer-generated rhythmic auditory stimuli (pitch 600 Hz) were administered through earphones (right ear), to pace the right heel strikes. IBIs were generated containing fractal Gaussian noise with corresponding scaling exponent (Hurst exponent *H*). Short audio samples of metronomes are available in Audio S1, and the Table S2 provides mean and standard deviations of IBIs series.

#### Tasks and procedure

We first determined the individual preferred walking speeds. The treadmill speed was increased every 10 s in steps of 0.1 km/h. When participants indicated that they reached their most comfortable speed (defined as “a speed at which you may continue walking for an hour”), the treadmill’s speed was further increased until participants indicated that the speed was too high. Then the speed was decreased until participants again indicated they reached their most comfortable speed again. The individual preferred walking speed was defined as the mean between the two speeds indicated by the participant, and used for all subsequent experimental conditions.

The experiment involved six walking conditions: self-paced walking (SP): participants walked at their previously determined preferred speed, without auditory stimuli (but wearing the earphones in order to create standardized conditions); isochronous metronome pacing with equidistant IBIs; and four fractal metronome pacing conditions with the scaling exponent of IBI series set at *H* = 0.9 (corresponding to very persistent fluctuations), and the coefficients of variation chosen as of *CV* 0.5%, *CV* 1%, *CV* 1.5%, and *CV* 2%. That range was chosen because the coefficient of variation of natural gait has been found to be lower than 3% [13], [25]–[26]. Each participant received individual metronome sequences. All sequences were generated using a fractional Gaussian noise generator with *H* = 0.9, meaning that the α-value of that sequence converges towards 0.9 for large samples sizes. The short sequences that we employed in the experiment had thus *α*-values distributed around the mean *H* = 0.9. Not using a single metronome sequence allowed for correlating IBIs and ISIs fractal exponents. For all pacing conditions the mean IBI delivered by the metronome was equal to the mean ISIs of each participant in the SP condition. Metronomes and recordings started at the same time, a few seconds after the treadmill was switched on and participants were walking. Each condition lasted six minutes yielding a sufficiently large number of strides (at least 256) to apply DFA after removing the first ten stride intervals to avoid transients [19].

Participants were explicitly instructed to synchronize the right heel strikes with the beats of the metronome and to maintain this synchronization as accurately as possible while keeping a natural and relaxed gait in case the metronome would present some variations. SP was the first condition for all participants; the order of the five pacing conditions was randomized. The same sampling frequency (300 Hz) was fixed for the generation of IBIs and force platform recording.

#### Data processing

Three main variables were analyzed: IBIs; ISIs, defined as the time intervals between two successive right heel strikes; and asynchronies to the metronome (ASYNs). These asynchronies were defined as the time intervals between right heel strikes and corresponding metronome beat onsets, so that negative asynchronies indicate anticipated heel strikes. The final 256 points of IBIs and ISIs were submitted to the subsequent DFA (time series available in Data S1). That is, next to the means and standard deviations of the collected time series our primary outcome measures were the *α*, *α*_short-term_, and *α*_long-term_ exponents, as explained above.

#### Statistics

To examine the ISIs correlation structure as a function of metronome conditions, we applied a 1×6 repeated measures ANOVA to *α* exponents. Tukey’s HSD was used for post-hoc analysis, and results were considered statistically different for *p*<0.05. To further assess the nature of adaptive processes occurring in fractal-pacing conditions, we tested for an individual matching of ISIs correlations to IBIs correlations using a linear correlation analysis between *α*_short-term_, and *α*_long-term_ exponents of IBIs and ISIs.

### Results

ANOVA revealed no significant differences in mean ISIs (*F*(5, 66) = 1.99, *p = *0.09), implying that the walking speed was largely constant across conditions. Also mean ASYNs did not differ significantly over conditions (*F*(4, 55) = 0.88, *p = *0.48). Mean values ranged from about −50 ms to about −40 ms (Table S1), that is, participants slightly anticipated the metronome as has often been observed in sensorimotor synchronization [27]. This suggests that in all conditions participants were able to adapt to the cueing.

**Figure 2:**
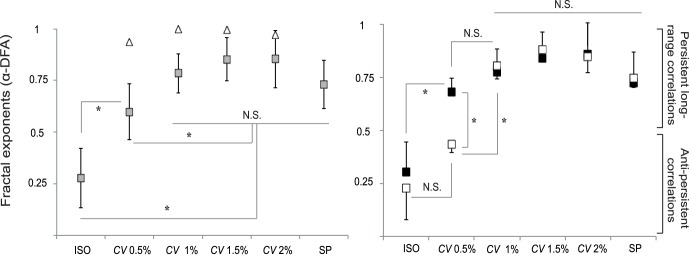
Mean *α* exponent (DFA) of inter-beat intervals (white triangles) and inter-stride intervals (squares) in Experiment 1. (*: *p*<0.05; N.S.: non-significant differences; error bars: standard deviation). *Left panel*: The evolution of *α* for all participants taken together (*n* = 12) could be interpreted as a progressive increase with increasing CV. *Right panel*: Qualitatively different changes in *α* exhibited by two subgroups: group 1 (black squares, *n* = 8) shows an abrupt switch from anti-persistent to persistent long-range correlations at *CV* = 0.5%, while group 2 (white squares, *n* = 4) switches at *CV* = 1%.

Mean *α* exponents obtained in IBIs and ISIs series are summarized in Table 1. For the ISIs series the ANOVA revealed a significant effect of pacing conditions on the global *α*-exponents (*F*(5, 66) = 36.34; *p*<0.001, Figure 2, left panel). Tukey’s HSD analysis indicated that *α* differed between SP and ISO conditions (*p*<0.001) with persistent long-range correlations for SP but anti-persistent correlations for ISO (mean *α* = 0.75±0.12 and *α* = 0.23±0.13, respectively). ISO was also significantly different from all of the fractal-pacing conditions (*CV* 0.5% to *CV* 2% all *p*<0.001). The mean *α* exponent in the *CV* 0.5% condition (mean *α* = 0.59±0.17) was close to white noise, and significantly smaller than those of all other fractal-pacing conditions (*CV* 1%, *p* = 0.004; *CV* 1.5%, *p*<0.001; and *CV* 2%, *p*<0.001).

**Table 1:** Mean fractal exponents (*α*_DFA_) and standard deviation (italics) of inter-beat intervals and inter-stride intervals estimated from all conditions in Experiment 1.

Experiment 1	ISO	*CV* 0.5%	*CV* 1%	*CV* 1.5%	*CV* 2%	SP
Inter-beat intervals (IBI)	–	0.94	1.00	0.99	0.97	–
	*–*	*0.11*	*0.10*	*0.11*	*0.10*	*–*
Inter-stride intervals (ISI)	0.28^a^	0.60^a^	0.78	0.85	0.85	0.73
	*0.14*	*0.13*	*0.10*	*0.10*	*0.14*	*0.12*

a p<0.05 when compared to SP condition.

In-depth analysis revealed however, that the mean *α* exponent obtained for *CV* 0.5% did not correspond to any of the individual series’ exponents. In fact the group showed a bimodal distribution: Eight participants switched from anti-persistent to persistent long-range correlations at *CV* 0.5%, whereas the remaining four participants switched at *CV* 1%. To address this bimodality we performed an additional 2(sub-groups)×6(pacing) ANOVA with repeated measures and found a significant effect of interaction between sub-groups and pacing conditions (*F*(5, 55) = 2.56; *p* = 0.038, see Figure 2, right panel). Tukey’s HSD analysis showed that in *CV* 0.5% the first sub-group produced long-range correlations in *CV* 0.5% (mean *α* = 0.69±0.11), which were qualitatively different from ISO (*p*<0.001) but not from other pacing conditions. The second sub-group produced anti-persistent ISIs (mean *α* = 0.40±0.02) that were qualitatively similar to correlations obtained in ISO pacing (*p* = 0.426). Post-hoc analysis confirmed that the two sub-groups differed only in *CV* 0.5% condition (*p* = 0.031).

**Figure 3:**
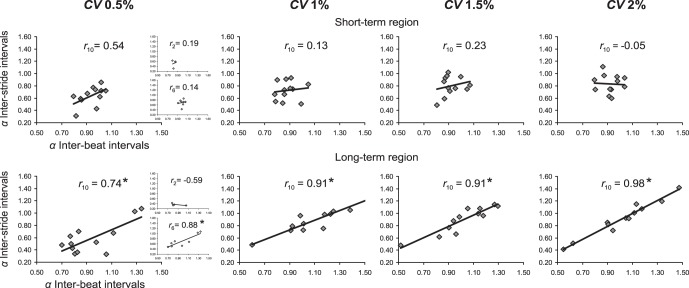
Correlation between *α* exponents (DFA) of inter-beat intervals and inter-stride intervals obtained in the four conditions with fractal metronome in Experiment 1. *Upper panel*: short-term region of diffusion plots. *Lower panel*: long-term region of diffusion plots. Significance threshold (*) for correlation coefficients is set at *p*<0.05 (*r*_10_ = 0.58). For the *CV* = 0.5% condition, subpanels separately show the correlations for the two subgroups of participants (*n* = 4, upper subpanel, and *n* = 8, lower subpanel).

The results of the linear correlation analysis on *α*_short-term_ and *α*_long-term_ exponents of IBIs and ISIs series are depicted in Figure 3. We found no significant correlation between *α*_short-term_ exponents for all fractal-pacing conditions (Figure 3, upper panel), while *α*_long-term_ exponents were positively correlated for all fractal-pacing conditions (*r_10_* ranging from 0.76 to 0.98, Figure 3, lower panel). This suggests that the observed complexity matching cannot be the consequence of the aggregation of short-term corrections.

## Experiment 2–Varying the Metronome Correlation

Our next step in investigating our participants’ ability to synchronize to fractal versus isochronous cueing was to compare different IBIs correlation structures for a fixed coefficient of variation. We expected that the fractal structure of ISIs would only be preserved for fractal-pacing conditions that agree with the correlation structure of self-paced walking. We therefore used metronomes with different IBI structures: anti-persistent, uncorrelated, and persistent.

### Methods

#### Participants

Twelve volunteers (five female, age 28±6 years) participated in the experiment after providing informed written consent. As in Experiment 1, all participants were healthy and none had any neuromuscular disorder or recent injury at the time of study. One participant of Experiment 2 also took part in Experiment 1.

#### Ethics

The ethics committee of the Faculty of Human Movement Sciences, VU University Amsterdam, The Netherlands, approved the experiment prior to its conductance.

#### Apparatus and equipment

The equipment was the same as in Experiment 1.

#### Tasks and procedure

The protocol was identical to Experiment 1 except for the use of different fractal cueing sequences. The conditions were: SP (see Experiment 1); ISO (see Experiment 1); and four non-isochronous cueing sequences with distinct scaling exponents, *H* 0.2, *H* 0.5, *H* 0.6, and *H* 0.9. In the latter four conditions, the IBIs coefficient of variation was fixed to 1%. Isochronous and non-isochronous conditions were sought to present the least differences. We thus chose the lowest *CV* in which we observed persistent fluctuations in ISIs for all participants in Experiment 1.

#### Data processing

Signal processing and estimates of IBIs, ISIs, and ASYNs agreed entirely with Experiment 1.

#### Statistics

We applied a 1×6 repeated measures ANOVA to all outcome measures. As in Experiment 1, Tukey’s HSD was used for post-hoc analysis. Results were considered statistically different for *p*<0.05. We also used a linear correlation analysis between *α*, *α*_short-term_, and *α*_long-term_ exponents of IBIs and ISIs.

### Results

Mean *α* exponents obtained in IBIs and ISIs series are given in Table 2. For ISIs series, ANOVA revealed a significant effect of pacing conditions on *α* exponents (*F*(5, 66) = 33.33; *p*<0.001). Tukey’s HSD analysis showed that *α* differed between SP and ISO conditions (*p*<0.001) with persistent long-range correlations for SP (mean *α* = 0.79±0.09) and anti-persistent correlations for ISO (mean *α* = 0.25±0.15). SP differed from the three non-fractal non-isochronous conditions (*H* 0.2, *H* 0.5, and *H* 0.6 all *p*<0.001). The *α* exponents in the *H* 0.9 condition differed from all other pacing conditions (ISO, *p*<0.001; *H* 0.2, *p*<0.001; *H* 0.5, *p = *0.004; *H* 0.6, *p* = 0.004), but not from the SP condition (*H* 0.9, *p* = 0.099).

**Table 2:** Mean fractal exponents (*α*_DFA_) and standard deviation (italics) of inter-beat intervals and inter-stride intervals estimated from all conditions in Experiment 2.

Experiment 2	ISO	*H* 0.2	*H* 0.5	*H* 0.6	*H* 0.9	SP
Inter-beat intervals (IBI)	–	0.23	0.52	0.57	0.91	–
	*–*	*0.04*	*0.03*	*0.08*	*0.15*	*–*
Inter-stride intervals (ISI)	0.25^a^	0.26^a^	0.44^a^	0.44^a^	0.64	0.79
	*0.15*	*0.11*	*0.13*	*0.09*	*0.17*	*0.09*

a p<0.05 when compared to SP condition.

**Figure 4:**
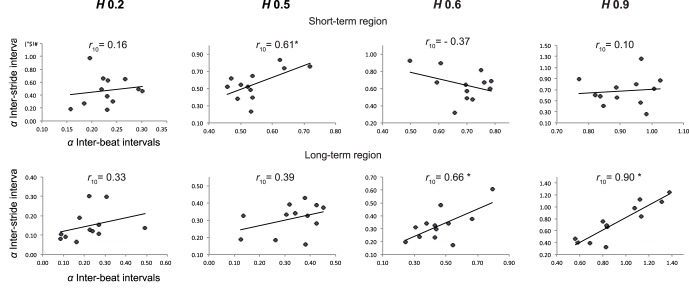
Correlation between *α* exponents (DFA) of inter-beat intervals and inter-stride intervals obtained in the four conditions with non-isochronous metronome in Experiment 2. *Upper panel*: short-term region of diffusion plots. *Lower panel*: long-term region of diffusion plots. Significance threshold (*) for correlation coefficients is set at *p*<0.05 (*r*_10_ = 0.58).

The results of linear correlation analysis on *α*_short-term_ and *α*_long-term_ exponents of IBIs and ISIs series in the four non-isochronous conditions are shown in Figure 4. The correlation between *α*_short-term_ exponents (Figure 4, upper panel) was significant only for *H* 0.5 (*r_10_* = 0.61*)*. The correlation between *α*_long-term_ exponents (Figure 4, lower panel) was significant only for *H* 0.6 (*r_10_* = 0.66) and *H* 0.9 (*r_10_* = 0.90). This result suggests a sensitivity of ISIs for the long-range structure of fluctuations of IBIs only when persistent fluctuations were present.

### Experiments 1 & 2– Revisited

In order to assess possible contributions of short-term behavioral correction to the matching of IBIs and ISIs long-term correlation properties, we further determined the cross-correlation between IBIs and ISIs series. Since the conventional cross-correlation is highly sensitive to the presence of persistent trends in the time series, which would lead to a systematic overestimation of the local co-variations between IBIs and ISIs [20], we used a windowed cross-correlation analysis (WCC): the cross-correlation between the first windows of 15 samples of IBIs and ISIs series was determined after locally detrending each series. These windows were shifted sample-by-sample yielding a series of *N*–15 cross-correlation coefficients at lag 0 (with *N* = length of the time series). The same procedure was repeated by considering different lags between IBIs and ISIs series, from lag −10 to lag 10 samples. We note that a significant cross-correlation coefficient at lag 0 would evidence that current ISI and IBI have similar lengths. The threshold of significance for 15-point windowed cross-correlation was given as *r*_13_ = 0.51 (*p*<0.05).

**Figure 5:**
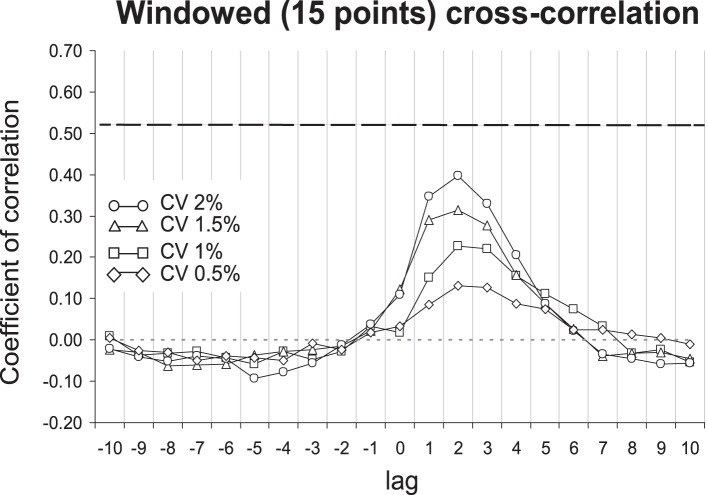
Mean coefficients of windowed (15-point window) cross-correlation functions (from lag −10 to lag 10) between inter-beat intervals series and inter-stride intervals series, for the four fractal-metronome pacing conditions in Experiment 1. The black dashed line shows the significance threshold (*r*_13_ = 0.5139, *p*<.05).

WCC revealed no significant correlation between IBIs and ISIs series in Experiment 1 (Figure 5). Overall, correlations increased with increasing variability (*CV*) in fractal IBIs with a maximum at lag two. In Experiment 2, WCC also showed no significant correlation between IBIs and ISIs series, and the maximum correlation was again found at lag two.

## Discussion

The findings of both experiments support our central hypothesis that when paced by a fractal metronome the correlation structure of stride intervals presents persistent fluctuations, contrary to isochronous cueing when the correlation structure of stride intervals presents anti-persistent fluctuations. We found a negative mean asynchrony in all cueing conditions, suggesting that participants were able to synchronize to the metronome irrespective of its correlation structure: even anti-persistent cueing could be followed. *α*_long-term_ exponents of ISIs and IBIs agreed only when IBIs presented fractal fluctuations implying that the persistent fluctuations in stride intervals were mainly influenced by the long-term correlations in the cueing.

In line with earlier reports [11]–[13] stride intervals exhibited persistent long-range correlations during self-paced walking and were anti-persistent during isochronous paced walking. The presence of long-range correlations is often considered a hallmark of healthy and adaptive complex systems [5], [28]. When the system is altered (by pathology or external constraints imposed on the subjects), deviations from this optimal behavioral dynamics may occur [8], [29]. In this respect, anti-persistence in stride intervals during isochronous pacing reveals a more rigid behavior: the gait dynamics is reduced to the regular tempo (single scale) of the metronome. For rehabilitation this may imply that patients only ‘learn’ to walk to the isolated cueing beat without the capacity to flexibly adapt to potential perturbations [16].

Stride intervals also presented anti-persistence when the IBIs presented anti-persistent or random fluctuations. Unlike isochronous, anti-persistent or random pacing, the use of a fractal metronome enables the locomotor system to maintain the fractal dynamics of gait, similar to self-paced gait dynamics (see Figure 2). The results of Experiment 2 suggest that this is due to the persistent, long-range correlations of inter-beat variations and not to the fact that the presented metronomes were merely variable (Figure 4). This agrees with recent findings that ISIs in elderly that listened to a chaotic metronome contained persistent correlations similar to those with no metronome [16]. According to Experiment 1, the fractal exponents of stride intervals between isochronous pacing and increasingly variable, fractal-pacing conditions were either anti-persistent or persistent, revealing that stride dynamics differed completely between isochronous and fractal pacing. Inter-individual differences in the *CV* 0.5% condition might be explained by differences in the perception of inter-beat variations of the metronome [29], [30].

The results in the fractal-pacing conditions (i.e., all conditions in Experiment 1, and *H* 0.9 in Experiment 2) revealed that, in general, the average fractal exponents of stride intervals tended toward the exponents of the inter-beat intervals presented. In both experiments this largely agreed with the exponents observed in self-paced stride intervals (Figures 2 & 4). In *H* 0.9 condition the mean fractal exponent decreased compared to self-paced condition. This might be due to individuals that produced anti-persistent fluctuations: a candidate, albeit speculative, explanation is that *CV* = 1% did not suffice for these participants (as *CV* = 0.5% was not enough for some others in Experiment 1). Hence, at first glance, participants could just have walked at their preferred speed without any effective synchronization to the auditory stimuli rendering this finding not necessarily indicative of adaptive behavior (like in [16]). In fact, synchronization with a fractal metronome (i.e. a largely unpredictable signal) appears quite counterintuitive. However, mean asynchronies obtained in the four fractal-pacing conditions in Experiment 1 and the four non-isochronous pacing conditions in Experiment 2 were similar to asynchronies obtained for isochronous pacing (≈ −40 ms, which is in line with earlier reports, e.g., [11], [27]), thus indicating effective synchronization with slight anticipation. Importantly, the CV of IBIs were below the mean asynchronies: the mean deviations were about 6, 12, 18 and 23 ms for CV 0.5, CV 1, CV 1.5 and CV 2, respectively. Consequently the negative mean asynchrony may have occurred by chance because participants simply adjusted the mean stride intervals to the mean beat intervals. If it was the case, however, we should not have observed any difference in the structure of stride intervals between isochronous and fractal pacing. Our results clearly demonstrate that the presence of fractal fluctuations in the metronome influenced the statistical signature of stride intervals.

Recent findings [31] underscored that the locomotor system cannot be ‘healthy’ by producing persistent fluctuations when self-paced and – by the same token – ‘unhealthy’ by producing anti-persistent fluctuations with isochronous cueing. Dingwell and co-workers [31] suggested that the diminution of the fractal structure in cueing conditions in elderly and in pathology might reflect an increase in stride-to-stride gait control (‘cautious gait’). Our results do not support this idea. We argue that participants controlled their strides at least as much (and maybe even more) with isochronous conditions as in non-isochronous conditions. In particular in Experiment 1, the metronomes with coefficient of variations of 2% were more variable so that we expected participants to be more careful to not miss any beats (as they were instructed to do so). Participants succeeded and asynchronies were about −50 ms, similar to those in isochronous conditions. However, ISIs were persistent (*α = *0.85, see Table 1) and similar to the self-paced conditions. That is, our results are not in favor of the hypothesis that fractal exponents decrease with increased control. We are aware that the results presented here are not sufficient to discard this interesting hypothesis and further studies should examine it in more detail. Simulations of stride intervals in non-isochronous conditions may indeed help to unravel the control mechanisms that are at work here. These numerical assessments are beyond the scope of the present paper but will be reported elsewhere.

Our results further provide evidence for a ubiquitous, strong correlation between the individual ISIs exponents and those of the corresponding IBIs series over the long-term region of the DFA plot (Figures 3 & 4). We consider this matching of fractal exponents a hallmark of strong anticipation. Strong anticipation is described as a global synchronization on a broad range of time scales instead of local step-to-step corrections to achieve synchronization [20]–[22]. We verified that the matching of the temporal correlation structures occurred mainly on the long-term part of the DFA plot: for the four fractal-pacing conditions of Experiment 1, our results showed very high correlations between DFA exponents of individual ISIs and IBIs series computed over the long-term region, but no significant correlation between exponents computed over the short-term region. Importantly, this correlation between DFA exponents on the long-term region occurred in Experiment 2 only when the IBIs series contained fractal long-range correlations (*H* 0.6 and *H* 0.9), but not in the case of anti-persistent correlations (*H* 0.2) or random fluctuations (*H* 0.5).

To our knowledge, the distinction between short-term and long-term regions of the diffusion plot represents an original methodological approach, which allows one to identify the relative contributions of local short-term and global long-term fluctuations to sensorimotor synchronization. To complement this analysis, we used windowed cross-correlation analysis, which specifically assesses the local co-variations of inter-stride and -beat intervals. This analysis showed no significant cross-correlation at any lag (from lag −10 to +10), nor in any of the four fractal-pacing conditions in Experiments 1 and 2. Synchronization with a variable stimulus has been described as “a mix of reaction, proaction, and synchrony” ( [22], p. 5274). Recall that the maximum (lag two) coefficient increased as the variance in the inter-beat intervals increased, suggesting that too much variability might involve more and more reactive processes. Taken together, we may suggest that persistence of the long-range correlated gait dynamics in fractal-pacing conditions mainly results from the matching of the long-range correlation structure of stride intervals performed onto the IBIs series presented.

Our interpretation strongly capitalizes on the estimated scaling exponents *α*, which brings us to the study’s major weakness: the brevity of pacing sequences profoundly limits the reliability of *α* estimates. DFA and related methods like the rescaled-range analysis, power spectral density estimates, wavelet-based scaling estimates, etc. all require a large number of samples, in particular if the expected (or generated) scaling exponents are larger than 0.5. The persistent long-range correlations do not only camouflage possible non-stationarities in the data but also cause a significant spread of *α* estimates. For instance, in the case of *H* = 0.9, numerical simulations of fractal Gaussian noise with *N* = 256 samples may yield standard deviations that can readily exceed Δ*α = *0.15. Although DFA appears to be the most reliable approach to assess the presence of long-range correlations [19], [32] we realize that our results should be interpreted with great caution although this lack of reliability is not necessarily reflected in the group statistics. We note that these considerations do not only apply to the estimates of *α* but also to those of *α*_short-term_ and *α*_long-term_. That is, future studies should aim for significantly longer pacing sequences, i.e. much longer walking protocols (preferably *N* ≥600 strides, see [33]). Given the plenitude of conditions in our experimental design, however, such extended protocols were not deemed feasible for answering our research questions.

Another limitation that should be mentioned is that the participants in the present study walked on a treadmill, which is known to be different from over-ground walking, putatively due to differences in the perceptual information generated. In particular, compared to over-ground walking, treadmill walking appears to be characterized by a less correlated pattern in the stride intervals and greater gait stability [25]. This could imply that the results of the present study would have been even more pronounced over ground; however, this remains speculation as long as a direct empirical comparison remains absent. Hence, also in this regard, the present results must be qualified.

In conclusion, the presence of long-range correlations in auditory cues enabled participants to maintain their ‘normal’, fractal gait pattern. This complexity matching of inter-stride intervals structures seemed to rely on strong anticipation processes with the attunement of ISIs and IBIs fractal exponents on the long-term region of DFA diffusion plots (supported by the absence of local cross-correlations). Our results may form a first step towards a better understanding of the effect of (correlations in) auditory cueing on gait. The present findings may open new opportunities for optimizing cueing protocols in gait rehabilitation. In particular, further investigations should verify a potential “carry-over” effect of a fractal metronome to stride-time dynamics.

## Supporting Information

Table S1 Means and standard deviations of the series of asynchronies (standard deviations in italics) from all conditions in Experiment 1 (upper panel) and Experiment 2 (lower panel).
(EPS)

Table S2 Means and standard deviations of the series of inter-beat intervals (standard deviations in italics) from all conditions in Experiment 1 (upper panel) and Experiment 2 (lower panel).
(EPS)

Audio S1 Audio examples of pacing signals (only 15 seconds). File names corresponds to the conditions (for example : Exp1_CV1 correspond to a fractal metronome used in Experiment 1, in condition *CV* = 1%).
(ZIP)

Data S1 Time series from Experiment 1 and Experiment 2 (.xls files). Each sheet correspond to a particular condition for a particular variable (for example : in the file « data_expe1_multipleCV », the sheet ISI CV 1% corresponds to stride-time series of participants in the condition CV = 1% in Experiment 1). Each column corresponds to an individual participant.
(ZIP)
